# Occurrence of Fibropapillomatosis in Green Turtles (*Chelonia mydas*) in Relation to Environmental Changes in Coastal Ecosystems in Texas and Florida: A Retrospective Study

**DOI:** 10.3390/ani12101236

**Published:** 2022-05-11

**Authors:** Costanza Manes, Daniele Pinton, Alberto Canestrelli, Ilaria Capua

**Affiliations:** 1 Department of Wildlife Ecology and Conservation, University of Florida, Gainesville, FL 32611, USA; 2 One Health Center of Excellence, University of Florida, Gainesville, FL 32611, USA; icapua@ufl.edu; 3 Department of Civil and Coastal Engineering, University of Florida, Gainesville, FL 32611, USA; daniele.pinton@ufl.edu (D.P.); alberto.canestrelli@essie.ufl.edu (A.C.)

**Keywords:** coastal waters, wildlife diseases, ecosystem change, environmental impact, marine ecology, turtles, viruses

## Abstract

**Simple Summary:**

In the last few decades, sea turtles have been threatened by a disease called Fibropapillomatosis. Infection causes the growth of several tumors which can prevent affected turtles from seeing, swimming, and feeding properly, often with lethal outcomes. Fibropapillomatosis was described for the first time in Florida in 1938 and has since then increased and spread worldwide. To this day, there is no strong nor clear evidence on what causes the exacerbation of this disease which is associated with a herpesvirus. There is a consensus, however, that human-driven changes in the sea turtle habitats (i.e., climate change, pollution, urbanization) might play a role in increasing the number and severity of clinical cases. This study intends to explore the role of various possible environmental drivers behind the increased occurrence of this disease. We found that sea temperature, salinity, human population density, and river discharge from the coastline could be important drivers of tumor prevalence. Results from this preliminary work have the potential to offer an important baseline for future research on environmental drivers of Fibropapillomatosis.

**Abstract:**

Fibropapillomatosis is a neoplastic disease of marine turtles, with green turtles (*Chelonia mydas*) being the most affected species. Fibropapillomatosis causes debilitating tumor growths on soft tissues and internal organs, often with lethal consequences. Disease incidence has been increasing in the last few decades and the reason is still uncertain. The potential viral infectious agent of Fibropapillomatosis, chelonid herpesvirus 5, has been co-evolving with its sea turtle host for millions of years and no major mutation linked with increased disease occurrence has been detected. Hence, frequent outbreaks in recent decades are likely attributable to external drivers such as large-scale anthropogenic changes in the green turtle coastal marine ecosystem. This study found that variations in sea surface temperature, salinity, and nutrient effluent discharge from nearby rivers were correlated with an increased incidence of the disease, substantiating that these may be among the significant environmental drivers impacting Fibropapillomatosis prevalence. This study offers data and insight on the need to establish a baseline of environmental factors which may drive Fibropapillomatosis and its clinical exacerbation. We highlight the multifactorial nature of this disease and support the inclusion of interdisciplinary work in future Fibropapillomatosis research efforts.

## 1. Introduction

Fibropapillomatosis (FP) is a neoplastic disease affecting all sea turtle species, with the green turtle (*Chelonia mydas*) being the most heavily affected [1]. FP was reported for the first time in Florida in 1938 and has since spread around the state and beyond, reaching worldwide occurrence [2,3]. Juvenile green turtles recruiting to coastal ecosystems, in particular, have been experiencing FP prevalence of over 50% in recent years [4,5,6]. Symptoms consist of debilitating tumoral growths on the turtle body (i.e., mouth, eyes, flippers areas, underside of shell), impeding basic survival activities such as feeding and swimming [1]. Sometimes lesions develop internally including on the lungs, kidneys, and the heart, with lethal consequences for the turtles [7]. The likely viral infectious agent of FP has been identified in chelonid herpesvirus 5 (ChHV5) [8]. ChHV5 has been evolving with its sea turtle host for millions of years, and no recent viral changes have been associated with FP incidence surge in the last few decades [9,10]. Thus, ChHV5 is not believed to be the only responsible agent inducing the disease, as the temporal change in prevalence points towards a multifactorial mode of inception. Post-pelagic green turtles are nearshore foragers and dwell in coastal waters [11]. In the last few decades, they have experienced gradual changes and alterations in their habitat [12,13]. This environmental disruption has coincided with consistent and increasing outbreaks of FP in the wild, disproportionately affecting the juvenile band of the population [1,14,15,16]. Green turtles are an endangered species and, together with poaching, human encroachment, and habitat loss, FP can be a major threat to their conservation [17,18].

Several seawater parameters have been studied in the FP literature. Thermal stress resulting from changing temperatures can alter immune response, making sea temperature variations a potential factor in FP infection dynamics [19,20,21,22,23]. Viral infections in ectothermic vertebrates are significantly affected by temperature changes [24]. In the last 50 years, the global increase of FP prevalence has been overlapping with an average temperature increase of a third of a degree in the ocean [25]. Salinity levels can also be a stressor for marine reptiles and dictate habitat suitability for sea organisms, such as benthic dinoflagellates present in macroalgae blooms [26]. Harmful macroalgae blooms such as red tides (*Karenia brevis*) could also play a role in FP outbreaks, occurring in nearshore habitats and identified for their negative impact on aquatic species [27]. Furthermore, FP has been often associated with anthropogenic disturbance. Sea turtles dwelling in urbanized coastal environments have repeatedly shown elevated FP prevalence [4,15,22,28,29,30,31]. Proximity to densely human-populated areas, alongside habitat loss, pollution, and nutrient discharge can cause stress on marine animals, commonly linked to immune system suppression and infectious disease outbreaks [32]. River discharge from densely populated areas accounts for high amounts of contamination and affects nearshore seawater composition [33]. FP researchers have already investigated seawater contaminants in FP-infected turtles across Hawaii [29,34], Australia [30], and Brazil [35], and green turtle tissues have generally been found to accumulate pollutants also across the Indo-Pacific [36], Caribbean [37], and Mediterranean region [38]. Some findings showed blood contamination correlations with FP viral infection, oxidative stress, and overall poorer health [30,35]. Water currents are also an important factor to consider, as they modulate water temperature and salinity fluctuations, as well as nutrient migration and algal blooms propagation [39,40,41]. Moreover, ChHV5 is likely to persist in seawater for hours or even days, and molecular evidence of horizontal transmission has been reported [42,43]. This indicates that FP may disseminate between individuals via viral shedding in seawater [1,8,42,44,45,46,47]. Water currents have previously been indicated to play a role in viral transmission, following the concept of viral dilution [48]. In infected areas, more frequent and stronger currents could regularly dilute water, decreasing viral presence. Conversely, stagnant water potentially allows a higher accumulation of viral particles, increasing infection risk [49]. Hence, we deemed water residence time to be another valuable avenue to examine when investigating FP dynamics.

In the current study, we research the role of multiple environmental factors by comparing FP prevalence in green turtles across Florida and Texas with environmental variables extracted from public datasets. Environmental features selected for our analysis include sea surface temperature, salinity, water currents residence time, human population density, riverine nutrient discharge, and red tide events. Our approach was to explore the role of potential environmental drivers in the occurrence of FP on the Southeastern United States coast over different spatiotemporal frames in the last few decades. Notwithstanding the limitations of an observational study on existing data, we seek new insights with the aim of providing a baseline for future research on FP environmental etiology.

## 2. Materials and Methods

### 2.1. Data Collection

#### 2.1.1. FP Prevalence Data

**Figure 1:**
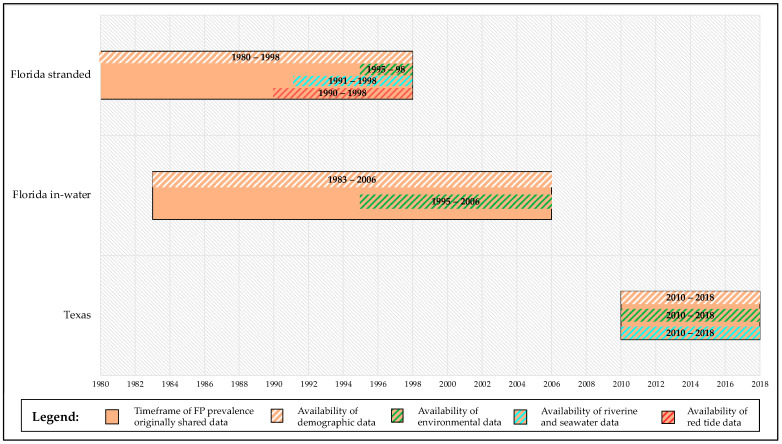
Timeframe of retrospective study. The orange blocks represent the timeframes of FP prevalence data shared by 3rd party authors or available in published papers for the different locations (1980–1998 for Florida stranded FP dataset [20]; 1983–2006 for Florida in-water FP dataset [50,51]; 2010–2018 for Texas FP dataset [14]). Matching timeframe of availability of predictor variables is reported for each group. Absence of predictor variables block in a given location (i.e., red tide data in Florida in-water locations) corresponds to NAs. Not all FP data could be utilized for the purposes of our study. Statistical analyses were run solely on the space and time matching timeframes from the earliest available timeframe after demographic data availability (i.e., patterns of Florida in-water FP prevalence *versus* environmental and demographic predictors from 1995 to 2006, see Section 2.2.1).

**Figure 2:**
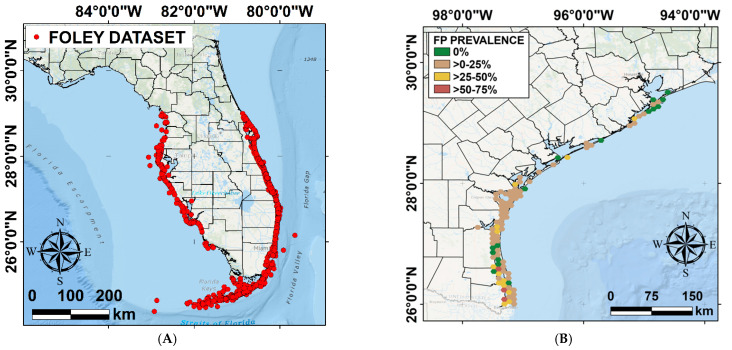
(**A**) Distribution of the surveyed turtles between 1980 and 1998 [20]; (**B**) distribution and prevalence of surveyed turtles affected by FP in Texas from 2010 to 2018 (adapted from Shaver et al., 2019) [14].

We conducted a literature search on the Google Scholar search engine (https://scholar.google.com/). Keywords used for the search were: [“Fibropapillomatosis” + “Green Turtle” + “*Chelonia mydas*” + “Prevalence OR Occurrence OR Incidence”]. We operated a selection of publications from the scientific literature according to their reporting criteria. Inclusion criteria selected papers that reported the following variables: (i) total number of FP-positive (FP_+ve_) individuals, (ii) total number of FP-negative (FP_−ve_) individuals, (iii) total number of individuals sampled, (iv) sampling location, and (v) year of sampling. The latter factor could not be a time range (i.e., 1986–1999), because annual FP prevalence was necessary to carry comparison with environmental and demographic data. To adjust for this factor, authors were contacted for permission to access their dataset. This narrowed the selection to four main publications that fit the scope of this research, three scientific articles and one published master thesis, reporting FP prevalence across various spatiotemporal frames (Figure 1). Foley et al. (2005) provided a detailed dataset of turtle stranding data across the Atlantic and Gulf coast of Florida from 1980 to 1998 [20] (Figure 2A). Hirama et al. (2014) and Borrowman (2008) provided a detailed dataset of in-water turtle captures from the Indian River Lagoon from 1998 to 1999 and across three different locations in East-Central Florida from 1983 to 2006, respectively [50,51]. Authors of these three sources directly shared the data collection broken down by year to allow for comparison in our research. Shaver et al. (2019) results also fit the scope of this research, reporting published data on FP prevalence from stranded or incidentally captured turtles along the Texas coast (Figure 2B), and were included in this study. The published paper already provided percentage data on FP prevalence broken down by location and year (from 2010 to 2018) [14]. Therefore, after the literature search and selection, our FP prevalence information comprised data from two United State states (Florida and Texas) and was divided into four categories: (i) Atlantic coast stranded Florida data, (ii) Gulf coast stranded Florida data [20], (iii) in-water Florida data [50,51], and (iv) Texas (stranded and incidentally captured) data [14]. Stranded data were divided between the two coasts as these can have different environmental characteristics, as also indicated by Foley et al. (2005) [20]. The in-water Florida data were only available for the Atlantic coast. In-water data indicates free-roaming individuals captured in the water with mesh tangle netting methods in the case of Hirama et al. (2014) and Borrowman (2008) [50,51]; or accidental captures (entrapped or by-catch) in the case of Shaver et al. (2019) [14]. While stranding data indicates individuals found stranded, dead, or alive but debilitated both in the case of Foley et al. (2005) and Shaver et al. (2019) [14,20]. Both publications also include dead or debilitated individuals found floating [14]. In our study, in-water and stranded individuals were not combined but analyzed separately, to avoid bias. The FP annual prevalence ratio formula was therefore applied to the separate FP datasets (stranded Florida Atlantic coast, stranded Florida Gulf coast, in-water Florida, and Texas) by calculating the resulting value from FP_+ve_ individuals sampled over total individuals sampled (i.e., FP_+ve_ + FP_−ve_) reported in each dataset for each year and location (i.e., 2 FP_+ve_/5 total sampled = 2/5 = 0.4 FP prevalence).

#### 2.1.2. Coastal Water Quality Data

**Figure 3:**
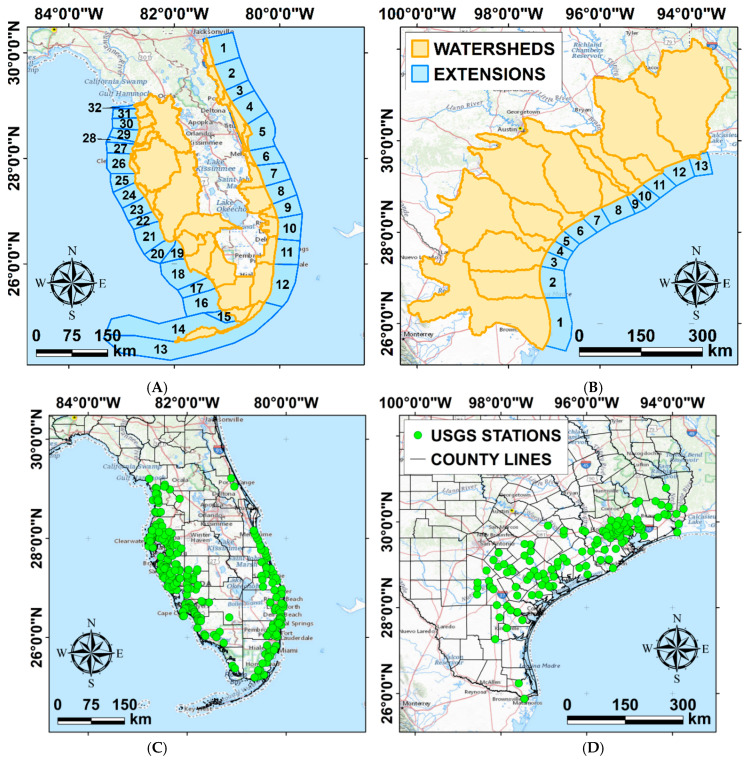
Distribution of the watersheds we identified in the study areas to divide the riverine water quality data and the population densities (orange polygons), and the areas we considered for the extraction of the environmental data from the HYCOM hydrostatic circulation model (blue polygons) in Florida (**A**) and Texas (**B**). Distribution of the USGS stations (green dots) in Florida (**C**) and Texas (**D**), where data was available in the considered timeframe (see Figure 1).

Water temperature, salinity, and residence time data were extracted from the Global Hybrid Coordinate Ocean Model (HYCOM) and the Navy Coupled Ocean Data Assimilation (NCODA) system. The HYCOM model evolved from the Miami Isopycnic Coordinate Model (MICOM). Its general architecture and validation are fully documented in Bleck (2002) and Halliwell (2004) [52,53]. HYCOM provides 4-day forecasts at 3-h time steps. To improve the initial condition of the forecast, the model assimilates available altimeter observations from satellites, in-situ, and satellite sea surface temperature, as well as in-situ vertical salinity and temperature profiles from XBT (expendable bathythermograph) instruments, Argo floats, and mooring buoys. The HYCOM model has a resolution of 1/12° (i.e., ~9.25 km) for both latitude and longitude. In the vertical direction, the properties of the water column (i.e., water depth, temperature, salinity, and flow velocity) are described by using 41 layers. The model uses isopycnal coordinates and conserves water and solute mass. It also provides high vertical resolution in weakly stratified regions, such as the surface mixed layer. The surface layer was chosen as, after their pelagic phase, green turtles recruit to neritic shallow-water habitats, and studies tracking diving patterns have observed green turtles spend most of their time (89–100%) within 5 m of water depth [11,54]. Data from 1995 to 2012 were extracted from the model reanalysis dataset (accessed 19 September 2020, from https://www.hycom.org/data/glbu0pt08/expt-19pt1). Data from 2013 to 2018, for which the re-analysis is not available, were extracted from the model forecast dataset (accessed 19 September 2020, from https://www.ncdc.noaa.gov/data-access/model-data/model-datasets/navoceano-hycom-glb). Sea surface temperature, salinity, and water residence time (eastward and northward directed) were extracted from 32 sites along the Florida coastline (blue polygons in Figure 3A) and 13 sites along the Texas coastline (blue polygons in Figure 3B). The values were calculated by averaging the model data in the sites. The areas were chosen to cover the zones where FP-affected turtle data were collected [14,20,50,51] and correspond to the ~50 km seaward prolongation of the considered watersheds. We chose 50 km to enclose the possible foraging area of the turtles in the seaward direction. Although aware of the generally restricted home ranges of green turtles, we operated the decision of including a wider extension inclusive of some of the largest ranges recorded for the species. Foraging home ranges as large as 39 km^2^ (3908 ha) have been recorded for green turtles as well as utilization of shallow coastal areas within 50 km offshore [55,56]. More research indicated centroid locations of core green turtle use area sometimes between 20–30 km from the shore in Florida waters [57]. Hence, we accounted for the chance that, even though mostly captured nearshore, turtles might have still been subjected to environmental conditions further offshore. Moreover, some of the original datasets also include some data points found further offshore [20]. Our estimate of 50 km was dictated by attempting to ensure to include all possible findings but also movements of green turtles within timeframes as long as the ones used for the analysis. Sea surface temperature and salinity were extracted only from the upper four vertical layers of the HYCOM model, which describe the upper 6 m of the water column, where green turtles have been observed to preferentially stay, avoiding the deeper waters [11]. Water flow velocity values were extracted from all the available layers. For shallow regions, less than four layers are available. Water velocity values were used to calculate the average residence time of the water column by dividing the volume of each cell by the integrated outflow of water. The latter was computed from 5-day mean water velocities at the boundaries [58]. As shown in Gray et al. 2019, the residence time depends on the extension of the area where it is calculated [59]. Note that, to be able to compare residence time between different areas, residence time was computed using the grid cells of the same size, i.e., the cells of the HYCOM model, and then averaged over the ~50 km seaward prolongation of the considered watersheds (blue polygons in Figure 3A,B). Finally, the annually averaged water temperature, salinity, and residence time values were calculated to be compared with the available annual FP prevalence in green turtles in the study periods and areas, as reported in Section 2.2. Water temperature, salinity, and residence time are reported as predictor variables in Table 1.

#### 2.1.3. Demographic Data

The population density (individuals per square kilometer) was calculated for the major watersheds we identified in Florida and Texas (orange polygons in Figure 3A,B) by dividing the total population of these watersheds by their surface area. Population density is reported as a predictor variable in Table 1. The major watersheds were identified by aggregating the watersheds in the study areas, extracted from the USA Watershed Boundary Dataset (accessed 22 February 2022, from https://landscape1.arcgis.com/arcgis/rest/services/USA_WatershedBoundaryDataset/MapServer), in 32 and 13 groups that contain the afferent area of the most important rivers that reach the Florida and Texas coastlines, respectively. Human population data were extracted from the Office of Economic and Demographic Research dataset (accessed 28 February 2022, from http://edr.state.fl.us/Content/population-demographics/data/index-floridaproducts.cfm) for Florida and from the US Census Bureau dataset (https://www.census.gov/) for Texas. Both the data sources provide the annual population estimates per county. The extracted temporal range was chosen to overlap the temporal range of the FP prevalence datasets on our study locations. Thus, ranges are from 1980 to 1998 for Florida stranded data (Figure 1, first row), from 1983 to 2006 for Florida in-water data (Figure 1, second row), and from 2010 to 2018 for Texas (Figure 1, third row). For each year, the total population ($P_{i}$) residing in the major watersheds, and its density ($D_{i}$), were calculated from the population estimates per county, as follows:

(1) $$P_{i}=\overset{j_{N}}{\underset{j=1}{\sum }}\frac{A_{j,i}}{A_{j}}P_{j},$$

(2) $$D_{i}=\frac{P_{i}}{A_{i}},$$

where $j_{N}$ is the number of counties (indicated as j), in which the area is partially or totally contained in the considered watershed (indicated as i), $A_{j,i}\;$is the area of the $j^{th}$ county, contained in the $i^{th}$ watershed, and $P_{j}$ and $A_{j}$ are the total population and area of the $j^{th}$ county.

#### 2.1.4. Riverine Water Quality Data

River discharge and water quality data were extracted from the United States Geological Survey (USGS) stations contained in the study areas in Florida and Texas (green dots in Figure 3C,D, accessed 26 February 2022, from https://maps.waterdata.usgs.gov/mapper/). Water quality data collected were river water discharge in m^3^·s^−1^, pH, and nutrient concentration (i.e., ammonia, chlorophyll, nitrite, nitrate, organic carbon, organic nitrogen, phosphate, orthophosphate, and phosphorus) in mg L^−1^. These predictor variables are reported in Table 1. Data were extracted for the years wherein FP prevalence datasets were available in the study locations in Florida and Texas. The USGS dataset presents temporal gaps, which vary depending on the location and the purpose of the stations. In particular, the data collected in Florida cover ~20% of the analyzed periods. This means that data are available for 20% of the corresponding years of FP data in Florida. The coverage value grows to ~30% for Texas. In addition, none of the USGS stations are located in watersheds 1, 2, 5, and 14, in Florida, and in watershed 2, in Texas. Once extracted, data were filtered to remove outliers, i.e., data whose distance from the mean is larger than ±5 times the standard deviation. Data were then divided between the watersheds indicated in Figure 3 (orange polygons). Once filtered and assigned to the watersheds, the mean annual values of the water quality parameters were compared with the yearly FP prevalence in the study periods and areas, as reported in Section 2.2.

#### 2.1.5. Red Tide Data

**Figure 4:**
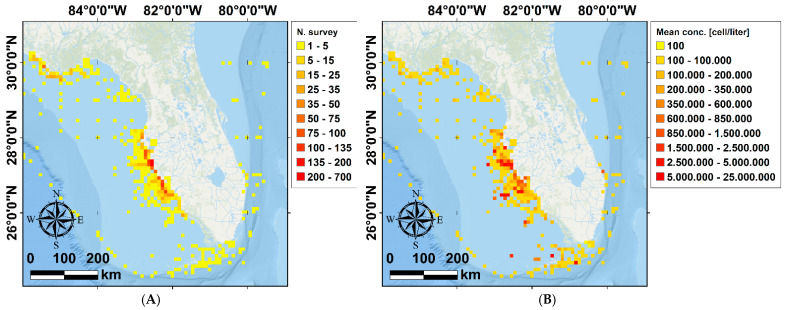
Heat maps representing the number of observations (**A**), and the average concentration in cells/liter (**B**) of *K. brevis* collected by FWC and FWRI (Fish and Wildlife Conservation Commission—Fish and Wildlife Research Institute) in the time frame where the environmental data were available (from 1987 through 1998, see Figure 1). The heat maps are represented using a regular grid. The dimension of the cells constituting the grid is 10 km × 10 km.

We accessed *K. brevis* abundance from the Florida Fish and Wildlife Conservation Commission–Fish and Wildlife Research Institute (FWC-FWRI) HAB Monitoring database (accessed 10 May 2020, from http://habsos.noaa.gov/), which contains the *K. brevis* 1954 to 2020 abundance data in Florida. To our knowledge, a dataset for Texas is not available. For Florida, data were collected by various private and public organizations using diverse sampling approaches, and therefore the temporal and spatial distribution of the data is neither systematic nor uniform. Indeed, the dataset has multiple gaps between 1954 and 2020 and does not homogeneously cover the Florida coastline (Figure 4). Moreover, many areas were only sporadically sampled, preferentially during large red tide blooms and depending on the availability of funding and projects, which is a limitation of this study. However, the highest density of available data was found along the western coast of Florida, between Tampa Bay and Gullivan Bay (Figure 4). This area also coincides with the maximum abundance of sea turtle data [20]. The annually averaged *K. brevis* concentration (cells L^−1^) and red tide occurrence (days) was calculated in the 32 locations along the Florida coastline (blue polygons in Figure 3A) and compared with the local data on FP-affected turtles. These predictor variables are reported in Table 1.

### 2.2. Statistical Analyses

#### 2.2.1. Spatial and Timeframes Selected for Analyses

As shown in Figure 1, this retrospective study often found data categories not matching in their spatiotemporal frames, due to the separate and different origins of each dataset. For the spatial frames used for the statistical models and analyses, the Florida Atlantic coast stranded dataset comprised 12 areas (polygons 1–12 in Figure 3A), and the Florida Gulf coast stranded dataset comprised 20 areas (polygons 13–32 in Figure 3A). This division was made consistently to the FP prevalence data classification by counties from the original dataset [20]. The Florida in-water dataset comprised 3 locations across 2 areas (polygons 5 and 7 in Figure 3A) [50,51]. The Texas dataset comprised 9 areas (polygons 1–9 in Figure 3B), while the original dataset reported FP prevalence divided across 3 main areas (North, Central, and South coast) [14]. For the purpose of our analysis, we mapped the spatial overlap between the original publication areas and the polygons, grouping them in the following way: polygons 1–3 for “North”, polygons 4–6 for “Central”, and polygons 7–9 for “South” (Figure 3B). For the timeframes used for the statistical models and analyses, demographic data were available throughout the whole timeframe of FP prevalence data availability in each dataset (Florida stranded—both Atlantic and Gulf coast, Florida in-water, and Texas). However, the analysis was run starting from the earliest matching timeframe after the demographic dataset, as running models from the start of the demographic timeframe would have included too many NAs (not available), confounding the factors and compromising statistical solidity. Thus, analysis for the Florida stranded dataset (both Atlantic and Gulf coast) was run from 1990–1998, analysis for the Florida in-water dataset was run from 1995–2006, and analysis for the Texas dataset was run from 2010–2018 (Figure 1).

#### 2.2.2. Analysis of Coastal and Demographic Datasets

Multiple linear regressions were run separately for the Florida Atlantic coast stranded dataset, Florida Gulf coast stranded dataset, Florida in-water dataset, and Texas dataset with FP prevalence as the response variable and four environmental and demographic factors (i.e., seawater temperature, salinity, residence time, and human population density) set as predictor variables.

#### 2.2.3. Analysis of Riverine Water Quality Dataset

Separate linear regression analyses were run to identify relationships between FP prevalence and the riverine water quality parameters reported in Table 1, collected in Florida and Texas. For the purpose of this particular regression model, stranded (Atlantic and Gulf coast) data were combined because of the paucity of the predictor variables sample size if separated (i.e., <5 data points), and the consequent low statistical robustness. Simple linear regressions were run using FP prevalence from the Florida stranded dataset and the Texas dataset, respectively, as response variables and with water pH, river discharge, suspended solids, and nine nutrients (i.e., ammonia, chlorophyll, nitrite, nitrate, organic carbon, organic nitrogen, phosphate, orthophosphate, and phosphorus) as predictor variables. No riverine water quality data were available for comparison with the Florida in-water dataset.

#### 2.2.4. Analysis of Red Tide Dataset

The red tide data are reported as the concentration (cells L^−1^) and occurrence (days) of *Karenia brevis* along the Florida coastline. Red tide data were not available for Texas, nor the Florida in-water dataset, and too small a sample size (i.e., <10 data points) for the Florida Atlantic coast stranded dataset. A multiple linear regression was run for the Florida Gulf coast stranded dataset using FP prevalence as the response variable and red tide concentration and occurrence as predictor variables.

For regression models analyzed between variables, Beta-estimates (*β*) and *p*-values (*p*) are reported. Following all regression models, Pearson’s correlation coefficients (*r*) were calculated between the response variable and each predictor variable separately for each of the four datasets, to assess the strength of each relationship. All linear regression models and Pearson correlation analyses were run via the statistical software RStudio (RStudio Team 2020. RStudio: Integrated Development for R. RStudio, PBC, Boston, MA. Retrieved from http://www.rstudio.com/).

## 3. Results

### 3.1. Multiple Linear Regression of FP Prevalence and Environmental and Demographic Factors

**Figure 5:**
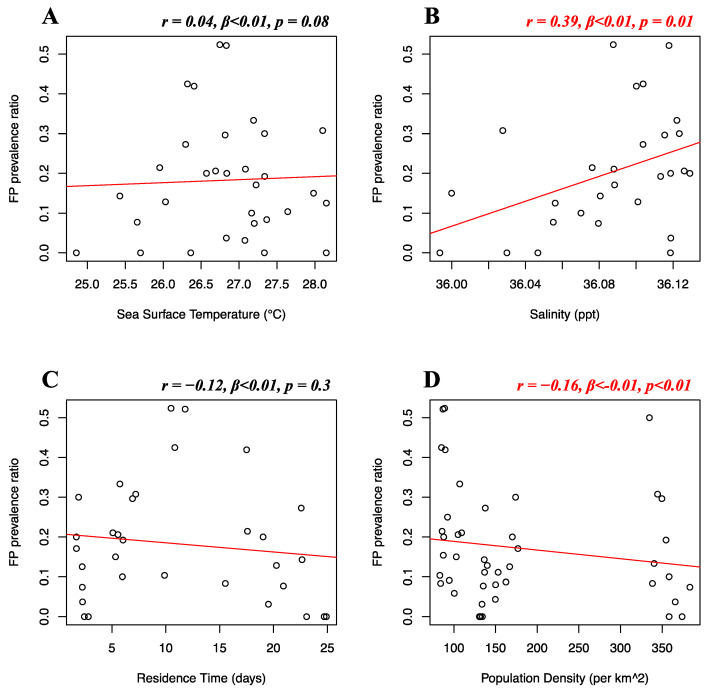
Florida Atlantic coast stranded dataset FP prevalence and environmental and demographic variables. Scatterplots with fitted regression lines representing the logistic regression output from the response variables versus predictor variables in the Florida Atlantic coast stranded dataset. The subplots show FP prevalence ratio on the *y*-axis versus sea surface temperature (**A**), salinity (**B**), residence time (**C**), and human population density (**D**) on the *x*-axis. Pearson correlation (*r*), beta-coefficient (*β*), and *p*-value (*p*) are reported for each predictor variable on the top right of the respective plot. Significant relationships are highlighted in red.

**Figure 6:**
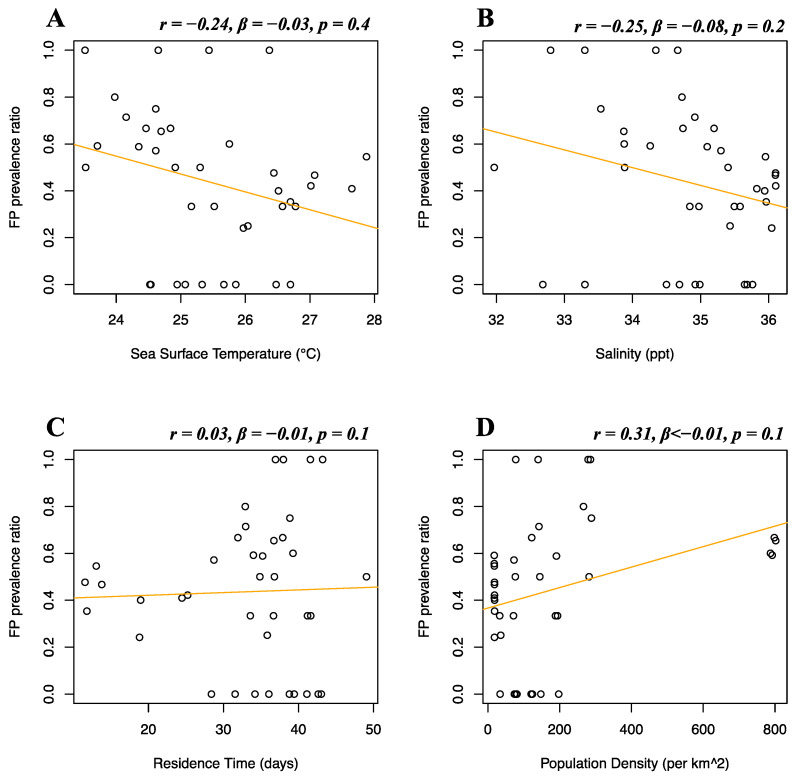
Florida Gulf coast stranded dataset FP prevalence and environmental and demographic variables. Scatterplots with fitted regression lines representing the logistic regression output from the response variables versus predictor variables in the Florida Gulf coast stranded dataset. The subplots show FP prevalence ratio on the *y*-axis versus sea surface temperature (**A**), salinity (**B**), residence time (**C**), and human population density (**D**) on the *x*-axis. Pearson correlation (*r*), beta-coefficient (*β*), and *p*-value (*p*) are reported for each predictor variable on the top right of the respective plot.

**Figure 7:**
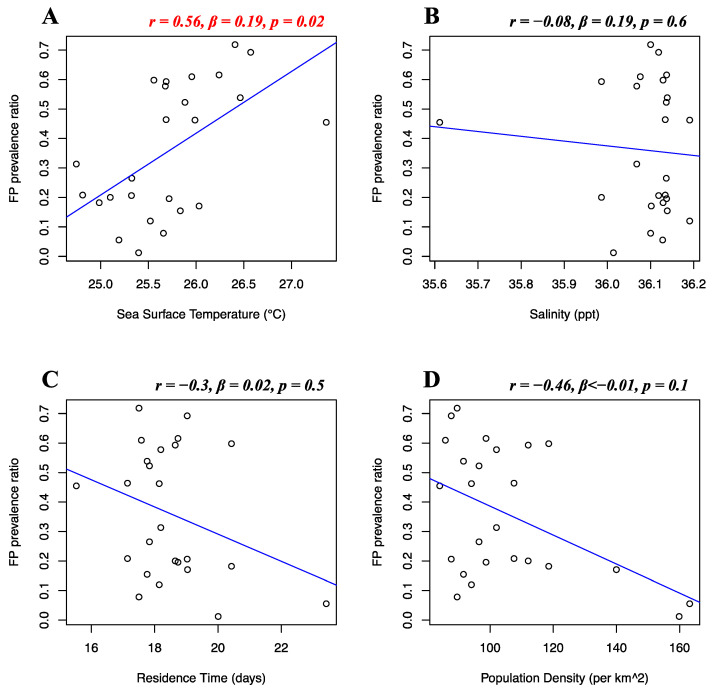
Florida in-water dataset FP prevalence and environmental and demographic variables. Scatterplots with fitted regression lines representing the logistic regression output from the response variables versus predictor variables in the Florida in-water dataset. The subplots show FP prevalence ratio on the *y*-axis versus sea surface temperature (**A**), salinity (**B**), residence time (**C**), and human population density (**D**) on the *x*-axis. Pearson correlation (*r*), beta-coefficient (*β*), and *p*-value (*p*) are reported for each predictor variable on the top right of the respective plot. Significant relationships are highlighted in red.

**Figure 8:**
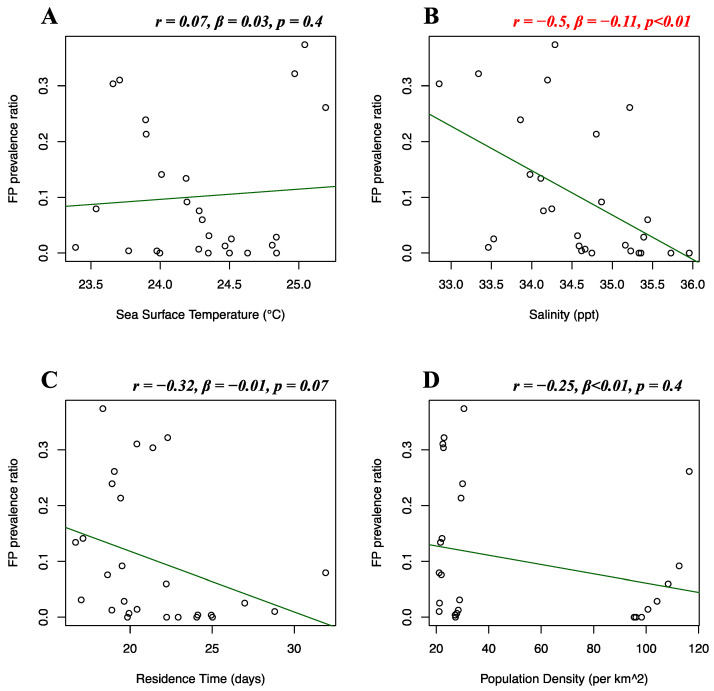
Texas FP prevalence and environmental and demographic variables. Scatterplots with fitted regression lines representing the logistic regression output from the response variables versus predictor variables in the Texas dataset. Subplots show FP prevalence ratio on the *y*-axis versus sea surface temperature (**A**), salinity (**B**), residence time (**C**), and population density (**D**) on the *x*-axis. Pearson correlation (*r*), beta-coefficient (*β*), and *p*-value (*p*) are reported for each predictor variable on the top right of the respective plot. Significant relationships are highlighted in red.

Florida Atlantic coast stranded data, Florida Gulf coast stranded data, Florida in-water data, and Texas data of FP prevalence were analyzed separately. Stranded and in-water data were not mixed when possible, as not considered comparable, given that these groups are likely biased in representing different levels of FP incidence [20]. For the Florida Atlantic coast stranded dataset, Florida Gulf coast stranded dataset, Florida in-water dataset, and Texas dataset, separate multiple linear regressions were run to test whether seawater temperature, salinity, residence time, and human population density significantly predicted FP prevalence. For the Florida Atlantic coast stranded dataset, it was found that salinity (*r* = 0.39, *β* < 0.01, *p* = 0.01) and human population density (*r* = −0.16, *β* < −0.01, *p* < 0.01) significantly predicted FP prevalence (Figure 5B,D), while seawater temperature (*r* = 0.04, *β* < 0.01, *p* = 0.08) and residence time (*r* = −0.12, *β* < 0.01, *p* = 0.3) did not significantly predict FP prevalence (Figure 5A,C). For the Florida Gulf coast dataset, it was found that seawater temperature (*r* = −0.24, *β* = −0.03, *p* = 0.4), salinity (*r* = −0.25, *β* = −0.08, *p* = 0.2), residence time (*r* = 0.03, *β* = −0.01, *p* = 0.1), and human population density (*r* = 0.31, *β* < −0.01, *p* = 0.1) did not significantly predict FP prevalence (Figure 6). For the Florida in-water dataset, it was found that seawater temperature significantly predicted FP prevalence (*r* = 0.56, *β* = 0.19, *p* = 0.02) (Figure 7A), while salinity (*r* = −0.08, *β* = 0.19, *p* = 0.6), residence time (*r* = −0.3, *β* = 0.02, *p* = 0.5), and human population density (*r* = −0.46, *β* < −0.01, *p* = 0.1) did not significantly predict FP prevalence (Figure 7B–D). For the Texas dataset, it was found that salinity significantly predicted FP prevalence (*r* = −0.5, *β* = −0.11, *p < 0*.01) (Figure 8B), while seawater temperature (*r* = 0.07, *β* = 0.03, *p* = 0.4), residence time (*r* = 0.32, *β* = −0.01, *p* = 0.07), and human population density (*r* = −0.25, *β* < 0.01, *p* = 0.4) did not significantly predict FP prevalence (Figure 8A,C,D).

### 3.2. Multiple Linear Regression Analysis of FP Prevalence and Riverine Water Quality

**Figure 9:**
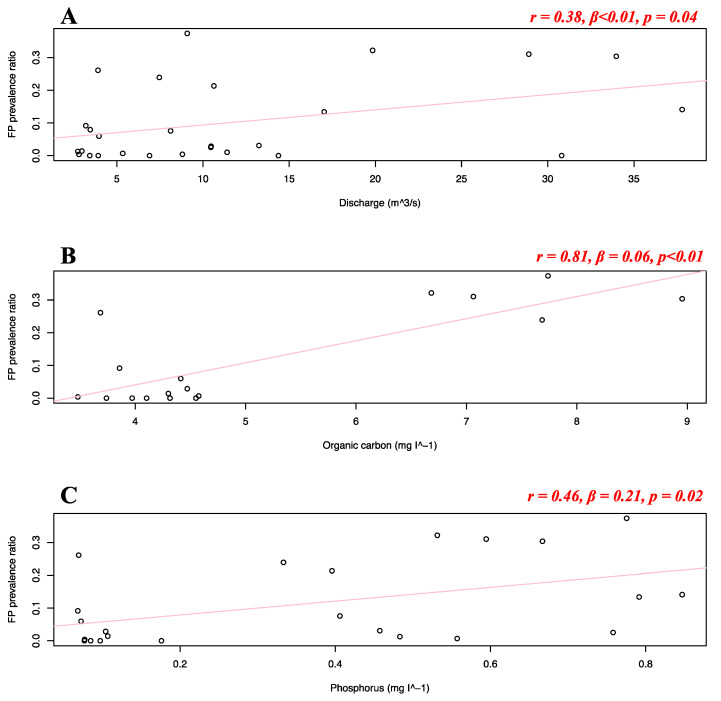
Texas dataset FP prevalence and discharge and nutrients variables. Scatterplots with fitted regression lines representing the logistic regression output from the response variables versus predictor variables in the Texas dataset: FP prevalence ratio on the *y*-axis versus discharge (**A**), organic carbon (**B**), and phosphorus (**C**). Pearson correlation (*r*), beta-coefficient (*β*), and *p*-value (*p*) are reported for each predictor variable on the top right of the respective plot. Significant relationships are highlighted in red.

Separate simple linear regressions were run to test if the analyzed seawater and riverine predictors (Table 1) significantly predicted FP prevalence in the Florida stranded dataset. For this analysis, FP stranded data from the Atlantic and Gulf coast were combined, as the availability of the predictor variables would be too small for statistical robustness in separate models (i.e., <5 data points). It was found that none of the riverine and seawater predictors significantly predicted FP prevalence in the Florida stranded dataset. Separate simple linear regressions were run to test if the analyzed seawater and riverine predictors (Table 1) significantly predicted FP prevalence in the Texas dataset. It was found that pH, ammonia, chlorophyll, nitrite, nitrate, organic nitrogen, phosphate, orthophosphate, and suspended solids did not significantly predict FP prevalence, while river water discharge (*r* = 0.38, *β* < 0.01, *p* = 0.04), organic carbon (*r* = 0.81, *β* = 0.06, *p* < 0.01) and phosphorus (*r* = 0.46, *β* = 0.21, *p* = 0.02) significantly predicted FP prevalence in the Texas dataset (Figure 9A–C).

### 3.3. Multiple Linear Regression of FP Prevalence and Karenia brevis Algal Blooms in Florida

**Figure 10:**
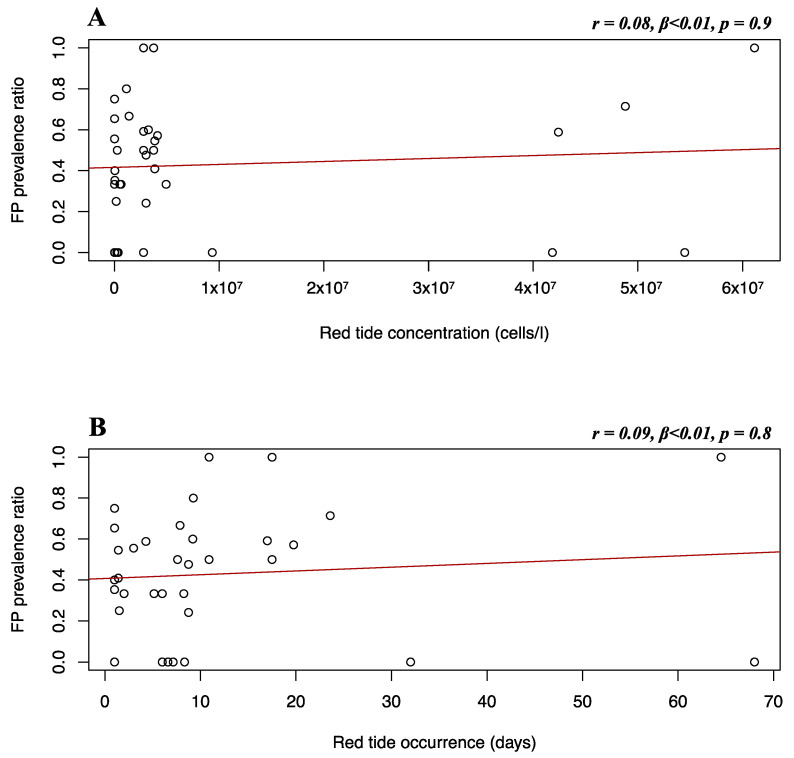
Florida Gulf coast stranded dataset FP prevalence and red tide variables. Scatterplots with fitted regression lines representing the logistic regression output from the response variables versus predictor variables in the Florida Gulf coast stranded dataset: FP prevalence ratio on the *y*-axis versus red tide concentration (**A**) and red tide occurrence (**B**) on the *x*-axis. Pearson correlation (*r*), beta-coefficient (*β*), and *p*-value (*p*) are reported for each predictor variable on the top right of the respective plot.

Due to limited sample sizes, *K. brevis* data were only available for comparison with the Florida Gulf coast stranded dataset. Multiple linear regression was used to test if *K. brevis* concentration and occurrence significantly predicted FP prevalence. Although a positive trend can be observed, it was found that *K. brevis* concentration (*r* = 0.08, *β* < 0.01, *p* = 0.9) and occurrence (*r* = 0.09, *β* < 0.01, *p* = 0.8) did not significantly predict FP prevalence in the Florida Gulf coast stranded dataset (Figure 10).

## 4. Discussion

This is an extensive integrative study aiming to understand the relationship between FP prevalence and a variety of environmental variables. Exploratory research on large pre-existing datasets often encounters limitations on data availability and quality and sample size, and this has been our experience as well. Despite the consensus and assumptions in the literature, not much is known about the specific role of selected environmental drivers of FP. Hence, we deem our results to be instrumental to develop a baseline of knowledge to direct future research avenues. Notwithstanding the aforementioned limitations, we demonstrate that sea surface temperature was a significant predictor of FP prevalence from in-water FP data from East-Central Florida (*r* = 0.56, *p* = 0.02) (Figure 7A). Variation in sea temperature has widely been indicated to affect FP prevalence in previous studies [20,23,60]. Thermal stress from temperature extremes can influence immune competence, triggering FP infection, proliferation, and viral shedding [19,21,61]. Our findings within our East-Central Florida in-water FP dataset support the hypothesis that seawater temperature may play a significant role in influencing FP prevalence in green turtle populations. We observed no clear pattern of FP prevalence increase over time in our Florida in-water dataset, nor a noticeable temporal increase for temperature values. This suggests that the observed relationship likely originates from a consistent pattern of period/areas of high FP prevalence in our dataset coinciding with high temperatures and vice versa, rather than a coincidental increment of variables over time. Temperatures in our Florida in-water dataset ranged from 24.7 °C to 27.3 °C, with an FP prevalence of 50–60% observed mostly around 26 °C. Studies witnessing higher water temperature effects on tumor growth of hospitalized sea turtles had a similar temperature range in rehabilitation tanks (23 °C to 27 °C) [60]. Further studies in rehab facilities observed the same pattern of a higher likelihood of green turtles’ FP development during the warmer months [22]. This pattern could be attributable to pathogenic behavior, often showing a trend of higher growth rate and reproductive output at higher temperatures [25]. In 1995, Herbst et al. observed that higher water temperatures experimentally promoted FP tumor growth, while lower temperatures delayed their onset [8,19]. This suggests that temperature-dependent exacerbation could be the case for ChHV5 behavior, with tumor proliferation occurring at higher temperatures. Another explanation behind the FP-temperature correlation could be attributed to green turtle metabolism across different seasons. A temperature-induced metabolic decrease has been documented in green turtles, with effects on growth rates and a pattern of higher tissue metabolic rates at higher temperatures [62,63]. A slower metabolism in the winter months might therefore contribute to less metabolic energy devoted to tumor growth rate, hence lower presence of tumors overall. Our results indicate that monitoring sea temperature and its correlation with FP may be of high importance, especially considering the rapidly progressing ocean temperature increase [63]. 

In addition, our study indicates a weak but significant (*r* = −0.16, *p* < 0.01) negative relationship between FP prevalence in the Florida Atlantic coast stranded dataset and human population density. This was unexpected, as anthropogenic disturbance was previously indicated as a potential co-driver of FP occurrence [4]. This result can be explained by the structure of our dataset. The adjacent Indian River and St Lucie counties are considered extreme data points, reporting our dataset’s highest FP prevalence (>50%), and lowest human population density (<90 individuals/km^2^). The area encompassed by these two counties has been reporting consistently high FP rates [64,65] and, although not highly populated, its coastal waters are subjected to elevated quantities of pollutants and residential runoff [4,16,66]. The dataset is then driven towards the opposite direction when representing the Miami-Dade area, which registers a population density approximately twice the rest of Florida. This pattern can clearly be observed in Figure 5D. A similar moderate correlation (although non-significant) is shown for population density in the Florida in-water data (*r* = −0.46), where the spatially restricted dataset might have contributed to this type of outcome (Figure 7D). Thus, watershed population density might be too indirect an indicator of actual anthropogenic disturbance. More direct indicators, such as pollution discharge from highly urbanized coastlines might yield more interesting and significant results, as supported by our study (Figure 9). We also found salinity levels to be a significant predictor of FP prevalence both in the Florida Atlantic coast stranded (*r* = 0.39, *p* = 0.01) and Texas (*r* = −0.5, *p* < 0.01) dataset, following opposite patterns (Figure 5B and Figure 8B). For our Florida Atlantic coast stranded dataset, the salinity range available for the analysis was extremely narrow (35.9–36.1 ppt) and the correlation coefficient weaker compared to the Texas model, hence the significance from the former regression might be less robust. Moreover, according to the riverine data, the Atlantic coast receives generally less quantity of freshwater inputs compared to the Texas coast, which is subjected to the discharge of multiple large riverine systems. We will, however, report that extreme salinity fluctuations have been observed to cause stress and immune system suppression in sea turtles [4,19,60]. Salinity levels have previously been observed to influence herpesvirus infection in other marine organisms, such as oysters [67,68]. A possible link between salinity and FP however has not been reported to the best of our knowledge and should be investigated further as a co-driver of clinical disease. Although the potentially disruptive effects of salinity fluctuations are known, there is no previous evidence of FP having a significant correlation with salinity levels. This is both interesting and counterintuitive as turtle physiology indicates that sea turtles are excellent osmoregulators, and slight fluctuations in salinity levels are unlikely to have serious disruptive consequences [69,70] and there are even records of green turtles found in freshwater environments [71,72,73]. However, salinity and river discharge are highly correlated, especially in the proximity of the river mouth, and lower salinity is inherently connected to increased freshwater runoff discharged in coastal marine waters [74,75]. Freshwater discharge in highly urbanized areas such as the Texas coast is likely to transport high concentrations of nutrients and pollutants. The negative relationship between FP prevalence in Texas and salinity might rather be a side effect, as it is consistent with the significant positive correlation we found between FP prevalence and river water discharge in our Texas dataset (Figure 9A).

For sea turtles, the negative effects of environmental contaminants from wastewater were already discussed in 1995, as research indicated environmental pollutants greatly impact turtle health [19,76]. Sea turtle populations living in heavily polluted coastal environments were repeatedly found to present elevated FP prevalence [15,22,28,29,30,31,50]. Research observed a substantially lower FP prevalence in open ocean sites compared to a coastal lagoon (Indian River Lagoon) which is heavily degraded by urban development and polluted from local drainage system inputs [4]. The Texas coastline, particularly the southern area, is the intersection point of wind-driven currents moving southward and northward, bringing pollutants and nutrients to this coastal area [77,78,79]. While the discharge range in Florida spans between 0 and 10 m^3^·s^−1^, the range for Texas was much higher with an average discharge of about 12 and a maximum of 38 m^3^·s^−1^. We used the novel approach of directly collecting hydrological data on river discharge and correlating it with FP prevalence along the Texas coastline (Figure 9A), thereby supporting the mainstream hypothesis in the FP literature on environmental triggers for the clinical disease. River discharge from urbanized areas has the potential to greatly disrupt the immune function of sea turtles, which are considered sentinels of marine ecosystem health [64]. The high nutrient and pollutant concentration that juvenile green turtles are subjected to upon recruitment to neritic areas is likely to elevate stress levels, suppress immune function, and possibly trigger the proliferation of ChHV5 and subsequent tumoral lesions. High discharge events (>10 m^3^·s^−1^) in our dataset temporally and spatially correspond to an FP prevalence of >30% (maximum recorded being 37%), while discharge events of <5 m^3^·s^−1^ to an FP prevalence of <10%. Along the Texas coast, the prevalence of FP has been significantly increasing in the last decade [14] and our research suggests that high river discharge from nearby watersheds could have played an important role. Organic carbon concentrations in Texas are also significantly correlated with the prevalence of FP disease (Figure 9B). Elevated concentrations of organic carbon sediments have previously been found to spatially overlap with areas of high FP prevalence (70%), while areas of low prevalence (20%) registered lower concentrations [80]. Our dataset has found a similar pattern with two main clusters, <5 mg I^−1^ and >7 mg I^−1^ corresponding respectively to patterns of low (>10%) and high (20 to 35%) FP prevalence (Figure 9B). Organic carbon stocks in the coastal environment are largely absorbed and trapped in seagrass meadows (*Thalassia testudinum*), the main diet of green turtles [81,82]. This makes them generally vulnerable to potential uptake of the sediment [80,83]. Organic carbon is generally good for ecosystems and a primary food source in marine food webs. However, its elevated sequestration could be indicative of ocean acidification (high CO_2_), which is characteristic of coastal areas [84], such as the Texas coast here analyzed [80]. Although our exploratory study represents a small fraction of the global FP burden, this finding is novel and of potentially high interest. If confirmed by further research, the role of carbon sediments in FP warrants further examination since ocean acidification is expected to increase in the future [84]. The significant link between FP prevalence and phosphorus concentration (Figure 9C) can also yield interesting questions. In 2008, the Texas coast experienced a dramatic event of harmful algal blooms, mainly composed of *Procrocentrum* spp. toxic dinoflagellate [85,86]. *Procrocentrum* spp. has been correlated to FP as it plays a role in the green turtle diet and naturally releases a known tumor promoter called okadaic acid, which depends strongly on environmental concentrations of phosphorus for production [26,87]. High phosphorus concentrations in Texas waters could stimulate okadaic acid production from dinoflagellates, which might in turn promote FP in local green turtle populations. Moreover, previous studies have found this nutrient to vary in blood chemistry values of green turtles at different stages of FP [88]. Although speculative and not currently under investigation, this hypothesis could indicate one possible role of environmental phosphorus on FP local dynamics. FP prevalence has significantly increased in Texas over the analyzed timeframe (2010–2018), as also reported in the original dataset publication [14]. Over that same period, the predictor factors resulting significantly impactful (namely salinity, discharge, organic carbon, and phosphorus) have not shown such a clear pattern of temporal increase, suggesting again that such variables were likely environmental co-drivers to FP prevalence in the sites and years here under analysis. For the remaining data, we believe that our results lacked statistical significance mainly due to limitations in our dataset and sample size (see Section 2.1.4). Our results indicate that future research could benefit from analyzing the impact of water quality on FP prevalence variation in green turtle populations.

Residence time is an incredibly valuable environmental avenue to look at when assessing FP infection risk in coastal green turtle populations. A higher residence time is an indicator of seawater stagnation, that favors the persistence of infectivity in water [48]. If currents within a water body are weak for several days, viral particles might accumulate and increase the infection risk for marine organisms dwelling in that area [49]. In addition to higher viral presence, prolonged stagnation in nearshore habitats promotes the accumulation of pollutants and nutrients from nearby urban areas, which lowers water quality [4,89]. Observation of higher FP prevalence in lower wave energy compared to more open areas has previously been reported [4,20]. Our retrospective approach found no significant relationships between water residence time and FP prevalence (Figure 5C, Figure 6C, Figure 7C, and Figure 8C) but, as the literature suggests, this factor could output interesting findings from more sophisticated research in the future, such as in-situ measurements of current velocity in FP-affected areas. We have observed no significant relationship between FP prevalence and red tide characteristics evaluated in our dataset. This may also be related to the fact that there is a large gap in the red tide dataset, inclusive of a large portion of NAs. Across our Florida Gulf coast stranded dataset, both red tide concentration and occurrence show a non-significant positive trend with FP prevalence (Figure 10). The lack of statistical significance was surprising, as the presence of macroalgal blooms has previously been reported to have sublethal and threatening effects on green turtle health, and the release of toxic components can be a source of stress and act as an immune system suppressant [27,90]. However, there are certain timeframe issues when analyzing red tide effects. It is highly difficult to estimate the period it takes for red tide blooms to affect disease development. Moreover, red tides have previously been associated with both seawater temperature [91] and pollution [92], as well as nitrogen-enriched water and river discharge [93]. Hence, we highlight the presence of a challenge in determining whether *K. brevis* is a direct health-disruptive effect, or a representation of low water quality, which in turn may facilitate FP development. We believe that our results lacked statistical significance mainly due to limitations in our dataset and sample size linked to the exploratory nature of our research.

## 5. Conclusions

Here, we present an extensively integrative retrospective study on FP environmental etiology. We have gathered FP data from the literature on green turtle populations across different coastlines of the southern United States and accessed large public hydrological and demographic datasets. We have then developed an original model to investigate the association between FP occurrence and selected seawater, riverine, and human disturbance parameters. Our findings support the hypothesis of environmental triggers behind FP and report significant effects of environmental and demographic factors in certain regions of Florida and Texas across the analyzed timeframes. Often datasets lacked acceptable sample sizes and had to be excluded from our analysis, hence our findings necessitate further field data. While recognizing the limitations of an exploratory retrospective study, it would be fruitful for future FP field-based studies to be comparably integrative. Throughout the FP data literature search, we have noticed a tendency not to report environmental variables. There is a recurrent focus on individual factors (i.e., age, size, gender, tumor score) in FP monitoring studies. Collecting and including environmental variables (i.e., water temperature, depth, salinity) could be crucial to expand future FP research, as this disease is clearly driven by multiple factors. Interdisciplinary research is pivotal when looking at complex conservation issues such as anthropogenic-exacerbated wildlife infectious diseases. In our study, combining wildlife conservation and data mining and expert analysis, we generated evidence that supports the need to integrate data on water characteristics and anthropogenic pollution in future studies on FP occurrence in sea turtles. Expanding research domains to include environmental drivers is key to understanding how the rapidly changing coastal habitats are influencing FP epidemiology worldwide. Such an approach will help in seeking novel solutions to mitigate future outbreaks of this debilitating tumor disease in marine turtles.

## Figures and Tables

**Table 1:** Water quality and hydrodynamics predictor variables contained in the table were used in the linear regression analysis to find a relationship with the FP prevalence values. The columns contain, in order, the name, the source, and the unit of measurement (U.M.) of the predictors.

Predictor Variables	Source	U.M.
Sea surface water temperature	HYCOM	[°C]
Sea surface water salinity	HYCOM	[ppt]
Water current based residence time	HYCOM	[days]
*Karenia brevis* concentration	FWC-FWRI	[cells I^−1^]
Red Tide occurrence	FWC-FWRI	[days]
Watershed population density	US Census Bureau	[individuals km^−2^]
River discharge	USGS	[m^3^·s^−1^]
Water pH	USGS	[–]
Ammonia (NH_3_ + NH_4_^+^)	USGS	[mg L^−^^1^]
Chlorophyll	USGS	[mg L^−^^1^]
Nitrite (NO_2_)	USGS	[mg L^−^^1^]
Nitrate (NO_3_)	USGS	[mg L^−^^1^]
Organic nitrogen	USGS	[mg L^−^^1^]
Total nitrogen (nitrate + nitrite + ammonia + organic nitrogen)	USGS	[mg L^−^^1^]
Phosphorus	USGS	[mg L^−^^1^]
Orthophosphate (PO_4_)	USGS	[mg L^−^^1^]
Organic Carbon	USGS	[mg L^−^^1^]
Suspended Solids	USGS	[mg L^−^^1^]

## Data Availability

FP data extracted and accessed from published articles were utilized while giving full credit to the original source (see Section 2.1.1). For Texas FP prevalence data, the dataset was accessed from D. J. Shaver, J. S. Walker, and T. F. Backof, “Fibropapillomatosis prevalence and distribution in green turtles *Chelonia mydas* in Texas (USA),” Dis. Aquat. Organ., vol. 136, no. 2, pp. 175–182, 2019, doi:10.3354/dao03403. For Florida FP prevalence data, datasets were accessed from 3rd Party Data: restrictions apply to the availability of these data. Data was obtained upon direct email contact from Allan Foley, Shigetomo Hirama, and Kelly Borrowman and are available from the original third-party authors with the permission of the original third-party authors. For environmental, demographic, coastal, riverine, and red tide datasets, data are available in publicly accessible repositories as reported in in-text links in Section 2.1.2. (for coastal water quality data), Section 2.1.3. (for Demographic data), Section 2.1.4. (for riverine water quality data) and Section 2.1.5. (for red tide data) in the manuscript.
